# Total laparoscopic partial hepatectomy versus open partial hepatectomy for primary left-sided hepatolithiasis: study protocol for a randomized controlled trial

**DOI:** 10.1186/s13063-023-07476-w

**Published:** 2024-02-22

**Authors:** Shu-bo Pan, Chun-li Wu, Da-chen Zhou, Qi-ru Xiong, Xiao-ping Geng, Hui Hou

**Affiliations:** Department of General Surgery, The Second Affiliated Hospital of Anhui Medical University, Furong Road 678#, Shushan District, Hefei, 230601 Anhui China

**Keywords:** Laparoscopic, Open liver resection, ERAS, Hepatectomy

## Abstract

**Background:**

The advantages of laparoscopic left-sided hepatectomy (LLH) for treating hepatolithiasis in terms of the time to postoperative length of hospital stay (LOS), morbidity, long-term abdominal wall hernias, hospital costs, residual stone rate, and recurrence of calculus have not been confirmed by a randomized controlled trial. The aim of this trial is to compare the safety and effectiveness of LLH with open left-sided hepatectomy (OLH) for the treatment of hepatolithiasis.

**Methods:**

Patients with hepatolithiasis eligible for left-sided hepatectomy will be recruited. The experimental design will produce two randomized arms (laparoscopic and open hepatectomy) at a 1:1 ratio and a prospective registry. All patients will undergo surgery in the setting of an enhanced recovery after surgery (ERAS) programme. The prospective registry will be based on patients who cannot be randomized because of the explicit treatment preference of the patient or surgeon or because of ineligibility (not meeting the inclusion and exclusion criteria) for randomization in this trial. The primary outcome is the LOS. The secondary outcomes are percentage readmission, morbidity, mortality, hospital costs, long-term incidence of incisional hernias, residual stone rate, and recurrence of calculus. It will be assumed that, in patients undergoing LLH, the length of hospital stay will be reduced by 1 day. A sample size of 86 patients in each randomization arm has been calculated as sufficient to detect a 1-day reduction in LOS [90% power and *α* = 0.05 (two-tailed)]. The trial is a randomized controlled trial that will provide evidence for the merits of laparoscopic surgery in patients undergoing liver resection within an ERAS programme.

**Conclusions:**

Although the outcomes of LLH have been proven to be comparable to those of OLH in retrospective studies, the use of LLH remains restricted, partly due to the lack of short- and long-term informative RCTs pertaining to patients with hepatolithiasis in ERAS programmes. To evaluate the surgical and long-term outcomes of LLH, we will perform a prospective RCT to compare LLH with OLH for hepatolithiasis within an ERAS programme.

**Trial registration:**

ClinicalTrials.gov NCT03958825. Registered on 21 May 2019.

**Supplementary Information:**

The online version contains supplementary material available at 10.1186/s13063-023-07476-w.

## Background

Hepatolithiasis is defined as a gallstone disease in the intrahepatic bile ducts and is mainly prevalent in Southeast Asia [1]. Hepatolithiasis may occur alone or with extrahepatic bile duct stones. Long-term hepatolithiasis can cause secondary biliary stricture, liver cirrhosis, and even cholangiocarcinoma [2]. Hepatectomy is one of the most effective methods for treating intrahepatic stones because it can simultaneously remove stones and relieve bile duct stricture [3, 4]. For hepatectomy of hepatolithiasis, due to the presence of stones in the left liver of most patients with intrahepatic bile duct stones, left hepatectomy, including left lateral hepatectomy and left hepatectomy, is the most common procedure performed. With the advancement of laparoscopic technology, laparoscopic liver resection has been used in the treatment of various liver diseases, including benign and malignant liver tumours [5, 6]. Since Louisville’s statement in 2008, the international status of laparoscopic hepatectomy has been widely recognized [7]. However, laparoscopic hepatectomy in patients with hepatolithiasis may be more difficult and challenging than open hepatectomy because patients with hepatolithiasis usually experience changes in their normal anatomy and perihepatic adhesion due to chronic inflammation [8–10]. To date, only a few retrospective studies have compared the outcomes of laparoscopic left-sided hepatectomy (LLH) with those of open left-sided hepatectomy (OLH) for hepatolithiasis, and the feasibility and efficacy of LLH have not been fully evaluated [11, 12].

Enhanced recovery after surgery (ERAS) programmes have been applied for patients receiving small-scale and large-scale liver resection [13, 14]. Several studies have shown that ERAS programmes are safe, feasible, and effective in reducing the median LOS in both open and laparoscopic resection [15, 16]. In some retrospective trials comparing earlier recovery or a reduction in LOS, perioperative evaluation or management has not been uniform [12, 17]. In addition, the value of laparoscopic left-sided hepatectomy for hepatolithiasis compared with that of open left-sided hepatectomy within an ERAS programme in terms of LOS and hospital costs has not been studied in a randomized controlled trial (RCT). However, randomized grouping of patients undergoing open or laparoscopic liver resection is dangerous. Due to the lack of clinical and patient equipoise of laparoscopic surgery, experienced centres and surgeons are reluctant to randomize patients. To take advantage of two centres’ preferences and disapproval of laparoscopic liver surgery to obtain a series of uninterrupted prospective patients, we constructed a randomized controlled trial design with two randomized groups (open and laparoscopic surgery) and a prospective registry. Combining an RCT with a prospective registry will improve the overall power and enhance the external validity and generalizability of the study results [18].

The day of discharge from the hospital is dependent on multiple factors, including patient expectations, local discharge logistics, cultural differences between countries, hospitals, and surgeons. LOS may therefore be considered an inappropriate endpoint for comparison of surgical interventions. Within the ERAS programme for liver surgery, a composite endpoint has been defined: time to functional recovery [15]. This endpoint, representing medical readiness for discharge, consists of clear and objectively measurable criteria. A patient is considered functionally recovered if they have a normal or decreasing serum bilirubin level, good pain control with oral analgesia only, tolerance of solid food, no intravenous fluid support, and independent mobility at the preoperative level [15]. Functionally recovered patients are generally capable of performing activities of daily living independently and are independent of hospital care. Therefore, we will conduct this randomized controlled trial to evaluate the safety and effectiveness of LLH for hepatolithiasis by comparing its clinical outcomes with those of OLH.

## Methods

### Design

A randomized clinical trial and a prospective registry with two parallel groups will be conducted at the General Surgery Department of the Second Affiliated Hospital of Anhui Medical University in China. The aim of this study is to compare the effect of two hepatectomy methods for hepatolithiasis by assessing factors related to mortality and morbidity, including the length of postoperative hospital stay (LOS), biliary leakage [19], posthepatectomy haemorrhage [20], posthepatectomy liver failure [21], incisional hernias, residual stone rate, and recurrence of calculus [22].

### Study population

Adult patients with an indication for elective left lateral sectionectomy and left hemihepatectomy because of left intrahepatic bile duct stones with irreversible disease, such as biliary strictures, severe parenchymal fibrosis, or atrophy.

### Inclusion criteria

To be eligible to participate in this study, a patient must meet all of the following criteria:

- Patients suitable for undergoing both laparoscopic left hepatectomy and open left hepatectomy of the liver
- Patients who can understand the nature of the study and its requirements
- Men and nonpregnant, nonlactating women between the ages of 18 and 80
- Patients with ASA I-II-III

### Exclusion criteria

Patients who meet any of the following criteria will be excluded from participation in this study:

- Inability to provide informed consent
- Patients associated with a tumour or who require bilioenteric anastomosis or left caudate lobectomy
- Immunodeficiency diseases, such as HIV
- Previous upper abdominal surgery (except for laparoscopic and open cholecystectomy)

### Randomization

Patient recruitment and the collection of written informed consent will be performed at the outpatient clinic. Patients will be randomized to the different groups using the block randomization method. Each block contains two groups: the OLH group and the LLH group. The block size will be hidden from the trial executors and clinicians. Possible balanced combinations of these groups within the block will be numbered consecutively. Then, blocks will be randomly chosen using the ‘randomize’ library of SPSS 22.0 (SPSS, Chicago, USA), and a series of randomly assigned OLHs and LLHs will be generated based on the random sequence of blocks. Allocation will be concealed using sequentially numbered, sealed opaque envelopes prepared by a member of our Clinical Trial Center. Patients will be randomly allocated to either OLH or LLH before surgery. The trial executors will receive randomly generated treatment allocations within sealed opaque envelopes. Afterwards, medical staff will personally inform the expert surgeon as to which treatment group the patient has been randomized. To avoid any potential prediction of group allocation, information on the block length will be kept away from the study site.

### Assignment of interventions: allocation

#### Sequence generation

A randomization table will be made by using the ‘randomize’ library of SPSS 22.0 (SPSS, Chicago, USA). The table will be managed by one staff member who is a doctor but not associated with this trial.

#### Allocation concealment mechanism

Eligible patients will be allocated to receive the designated intervention during the operation. However, they will get to know their allocation after POD3. On the other hand, the operator will be unblinded just before surgery to conduct the appropriate intervention for the patient.

#### Implementation

One designated staff member will generate the allocation sequence using the randomization programme. After research investigators obtain informed consent from the subject, the staff will assign the patient to the determined group. The operator and other related investigators will be blinded just before surgery to perform proper management.

### Trial interventions

#### The LLH procedures

**Fig. 1:**
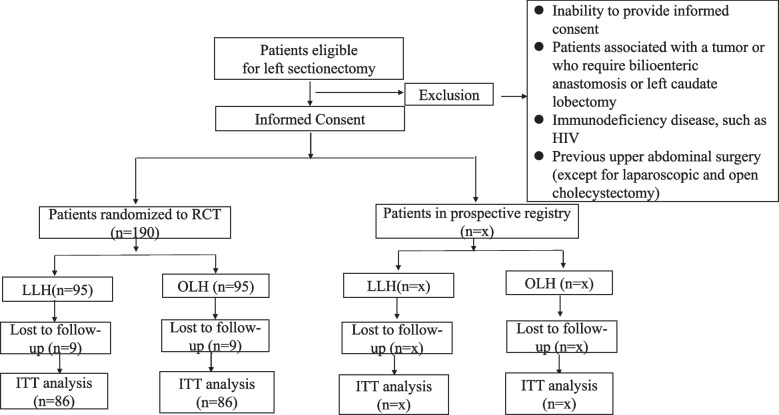
Surgical techniques for LLH

The LLH operative procedures will be completed by the same surgical team in each group led by two experienced board-certified surgeons. LLH and left lateral segmentectomy will be performed with the patient in the French position, with two experienced surgeons at each side (bilateral two chief surgeons) and a video laparoscope operator between the legs, which is different from the classic laparoscopic technique. Laparoscopic operations will be performed using 5 trocars. Common bile duct exploration (CBDE) will be performed in all patients with suspected stones in a dilated common bile duct (> 10 mm) using a choledochoscope, followed by T-tube placement if postoperative cholangiography is required [12] (Fig. 1).

#### The procedures of OLH

OLH will be performed under general anaesthesia with the patient in the supine position. An inverted L-shaped incision will be performed. The left hepatic artery and left portal vein will be individually dissected, ligated, and divided. Then, the left bile duct and left hepatic vein will be ligated, divided, and closed after hepatic parenchymal resection. The CBDE will be the same as the LLH.

#### Conversion from LLH to OLH

Any incision used for reasons other than trocar placement or specimen extraction will be defined as a conversion. Patients allocated to LLH but converted to OLH will still be analysed in the LLH group, according to intention-to-treat principles.

### Blinding

In this trial, all assessors of the primary outcome (i.e. the ward doctor), the patients, and the adjudication committee will be blinded to the operation allocation. Directly after skin closure, while still under general anaesthesia, patients will receive a firmly taped, large 40 × 40 cm abdominal dressing to cover their incision(s) and therefore their treatment allocation (minimally invasive or open). This abdominal dressing will be removed when all criteria for functional recovery are met or earlier for medical reasons, such as suspicion of wound infection. If an earlier inspection is needed, attempts will be made to maintain patient blinding.

The success of blinding will be assessed using the blinding index as proposed by Bang et al. [23]. Both patients and ward doctors will be asked about the alleged treatment allocation based on five categories: (1) strongly believe it was LLH, (2) somewhat believe it was LLH, (3) somewhat believe it was OLH, (4) strongly believe it was OLH, and (5) do not know. Sensitivity analysis will be performed for the analysis of time to functional recovery.

### Type of trial

The trial is a prospective randomized interventional single-centre study comparing two parallel groups with a prospective registry to determine whether patients underwent LLS by laparoscopic surgery rather than open surgery with an enhanced recovery programme. The types of interventions by patients, nurses, and ward doctors (not the surgeons performing the operations) before postoperative day (POD) 3 will be blinded. However, patients who are randomized to receive open or laparoscopic liver resection are at risk, as explained earlier. In addition, there is another potential source of bias when randomly selecting patients with strong treatment preferences. When patients are not informed about their treatment allocation (POD 3), they may be resentful and feel demoralized if they do not receive their preferred treatment. Thus, their compliance may be poor. In contrast, patients receiving their preferred treatment may have above-average compliance. Thus, to capitalize on centres both with and without preference for laparoscopic liver surgery and to acquire an uninterrupted prospective series of patients, all nonrandomized patients undergoing LLS will be approached to participate in the prospective registry. Registration of these patients is imperative to guarantee a consecutive series of patients and because the absence of such a series may restrict the generalization of the results, as randomized participants may not, in fact, be representative [24].

Nonrandomized patients will be asked for permission to use their data. In doing so, they will be assigned to the OLH or LLH group of the prospective registry on the basis that this registry might increase the external validity of results obtained in the randomized study [15, 18, 25].

### ERAS programme

**Fig. 2:**
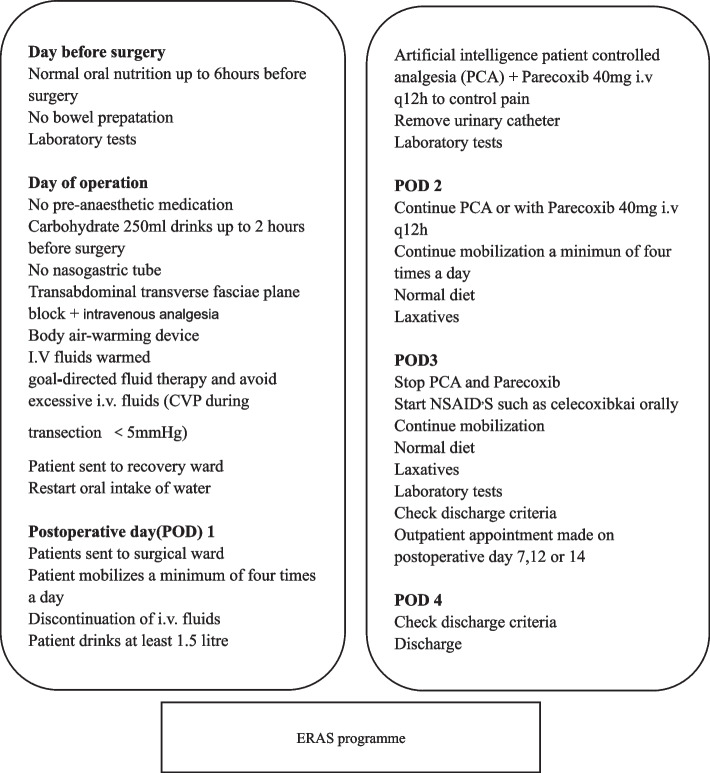
ERAS programme. CVP, central venous pressure

All patients will participate in the ERAS liver programme with standardized perioperative management. For the daily guidelines on the pre- and postoperative care of patients undergoing liver resection, see Fig. 2.

### Withdrawal

Patients can withdraw from the trial at any time at their own request or at the request of their legal representative. Patients may be removed if, in the researchers’ opinion, continuing the trial may be detrimental to the patient’s health, if left hepatectomy is not performed due to a technical inability, or for other reasons. Every withdrawal will be recorded in the clinical report forms (CRFs) and in the patient’s medical case records. All examinations scheduled for the final trial day will be performed on all patients and documented. All data will be analysed according to the intention-to-treat (ITT) principle [26].

### Primary endpoint

The primary outcome of this trial is the LOS. Only the researchers and surgical surgeons will know the true operation. A blinded ward doctor will decide whether the patient should be discharged.

### Secondary endpoints

The secondary outcomes are biliary leak [19], haemorrhage [20], posthepatectomy liver failure [21], wound infection, intra-abdominal fluid collection or abscess, relaparotomy, mortality, long-term abdominal wall hernias, hospital costs, residual stone rate and recurrence of calculus [22]. Existent ISGLS definitions will be used to ensure the comparability and generalizability of the results. Postoperative complications will be graded based on severity according to the Clavien–Dindo definition [27] (Tables 1 and 2).

**Table 1:** Definition of the secondary endpoints

Secondary endpoint	Definition and assessment of outcomes
Operative time	Time from beginning to end of operation
Mortality	Death due to any cause until 90 days after the operation and the reason
Morbidity	Postoperative complications will be recorded until 90 days after operation. The severity of complications will be graded according to the Clavien–Dindo classification [27]
Blood loss	Total blood loss during the operation
Intraoperative blood transfusion	Massive haemorrhage with hemoglobin level < 7 g/dl or Hct < 22
Postoperative hospital stay	Time from the day of operation until discharge(days)
Post-hepatectomy haemorrhage	Evidence of blood loss from drains and/or nasogastric tube, based on ultrasonography, as defined by ISGPS (grade A, B, or C) [20]
Biliary leak	Bilirubin concentration in the drain fluid at least three times the serum bilirubin concentration as defined as ISGLS (grade A, B or C) [19]
Post-hepatectomy liver failure	The impaired ability of the liver to maintain its synthetic, excretory, and detoxifying functions, which are characterized by an increased international normalized ratio and concomitant hyperbilirubinemia (according to the normal limits of the local laboratory) on or after postoperative day 5 as defined as ISGLS (grade A, B, or C) [21]
Intraabdominal fluid collection	Collection of fluid measuring ≥ 3 cm associated with clinical or laboratory abnormalities
Pneumonia	Presence of a new infiltrate on chest X-ray, as well as following: body temperature > 38℃, abnormal elevation of WBC, or positive sputum, and requiring antibiotic treatment
Wound infection	Surgical site infection associated with laparotomy that develops during the initial hospital stay
Abdominal rupture	Dehiscence of abnormal closure with need for resuture of the laparotomy during the initial hospital stay
Total hospitalization expenses	Hospital costs from admission to discharge ($)
Recurrence of calculus	Calculi in the intrahepatic duct within 3 months after hepatectomy are defined as residual stones [22]

**Table 2:** Severity grade according to the Clavien–Dindo definition [27]

Grade	Definition
I	Any deviation from the normal postoperative course without the need for pharmacological treatment or surgical, endoscopic, and radiological intervention
II	Requiring pharmacological treatment with drugs other than those allowed for grade I complications
III	Requiring surgical, endoscopic, or radiological intervention
IIIa	Intervention not under general anaesthesia
IIIb	Intervention under general anaesthesia
IV	Lifer-threatening complication. Requiring intensive care unit management
IVa	Single-organ dysfunction
IVb	Multiorgan dysfunction
V	Death of patient

### Data collection and patient follow-up

Data will be collected prospectively for all patients, including history, physical examination, laboratory data, pathologic examination, perioperative clinical information, and complications. Data will be collected via datasheets on paper and kept securely. Study subjects will complete the examinations at inclusion, 1 week after surgery, and 3, 6, 12, 24, and 36 months postoperatively. Follow-up will be conducted at 1 month postoperatively and then 3, 6, 12, 24, and 36 months postoperatively by telephone or as outpatients with liver function tests and abdominal ultrasonography for 3 years or until death. CT or MRCP will be performed in cases of suspected stone recurrence or cholangitis (Table 1). All handling cases will be managed by subject identification codes or anonymized registration numbers. The correspondence table of the anonymizing code and names and the consent form containing the names will be kept strictly in separate lockable document storage. All required parameters will be collected in an SPSS data file (SPSS version 25, IBM statistics, Chicago, IL, USA).

### Definitions

Functional recovery is reached when all of the following criteria are met: adequate pain control with only oral analgesia, restoration of mobility to a level of independence, ability to maintain sufficient caloric intake (a minimum of 50% of the required daily intake), no need for intravenous fluid administration, and no signs of active infection (fever or other clinical symptoms) [15]. Complications will be classified using the Clavien–Dindo score [27]. Major complications will be defined as a Clavien–Dindo grade III or higher.

### Quality and safety

The surgical experience of surgeons can affect complication rates, which might bias the results. To prevent surgeon bias, surgeons should meet the following criteria: (1) have performed more than 200 open hepatectomies, (2) have performed more than 100 laparoscopic hepatectomies, and (3) be qualified surgeons according to the China College of Laparoscopic Hepatectomy. LLH will be performed by two surgeons (Hui Hou and CL Wu), and OLH will be performed by four surgeons (Hui Hou, XP Geng, SX Xie, and QR Xiong).

Serious adverse events that have to be reported to the study coordinator within 24 h include unplanned intensive care unit admission; any surgical, endoscopic, or interventional radiology intervention (excluding feeding tube placement); readmission; and mortality (regardless of cause).

### Statistical aspects

#### Sample size calculation

**Fig. 3:**
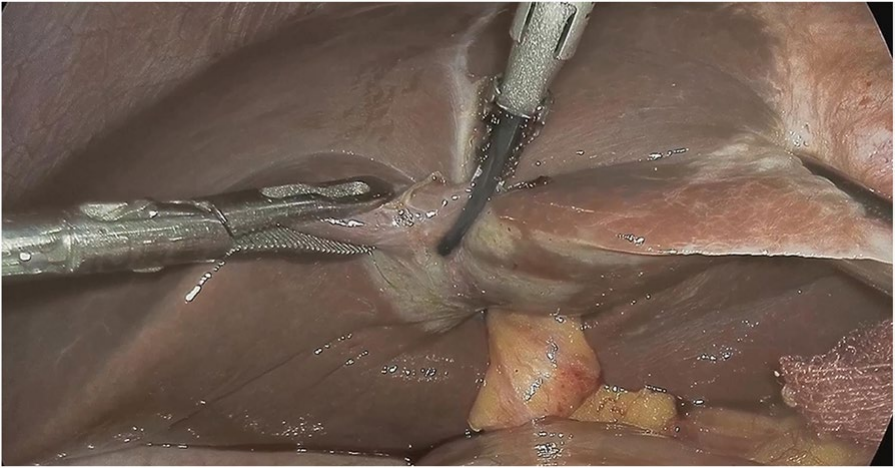
Flow chart according to CONSORT. X means there is no fixed number and that patients would recruit until the randomized element is full

The sample size calculation is based on the primary endpoint: the LOS. According to published data, an assumed 1-day reduction in LOS is the appropriate basis for the calculation, assuming 5 days in the LLH group and 6 days in the OLH group [28]. This calculation yields a total of 86 patients in each group, which assures 90% power at a two-sided level of significance of 5% [NCSS and PASS 15 (NCSS Statistical Software, Kaysville, UT, USA)]. Assuming an expected withdrawal rate of 10% during the trial, 18 additional patients will be included and randomized; therefore, the total sample size required is *n* = 190 patients (Fig. 3).

#### Statistical analysis

The two-sided null hypothesis for the primary endpoint states that both study interventions will lead to a similar time to the LOS; the alternative hypothesis is that one intervention will perform better than the other. The null hypothesis will be tested by analysing the covariance while adjusting for age and the type of liver resection. A binary logistic regression will be applied to compare the time to functional recovery between groups after adjusting for other factors. Background characteristics and surgical outcome measures will be compared using chi-squared or Fisher’s exact tests for categorical data and two-tailed* t* tests or nonparametric Mann–Whitney *U* tests for continuous variables. Categorical data will be presented as the frequencies and group percentages, and continuous variables will be expressed as the means and standard deviations. The homogeneity of the two groups will be described by comparison of the demographic data and baseline values. All analyses will be performed on an ITT basis [17]. For the ITT analyses, the data will be processed for all trial patients in their randomized groups. A *P* value < 0.05 will be considered statistically significant. All statistical calculations will be performed using SPSS 22.0 (SPSS version 25, IBM statistics, Chicago, IL, USA).

### Frequency and plans for auditing trial conduct

The principal investigator will submit an interim report every 12 months, and auditing will be conducted annually by designated members of the Medical Research Collaborating Center (MRCC) from the Second Hospital of Anhui Medical University. The auditing process will be independent of the investigators and the sponsor.

### Dissemination policy

The results of this trial will be submitted to a peer-reviewed medical journal regardless of the study outcome. The authorship will be based on international guidelines. Those involved with the study who do not fulfil these criteria will be listed as ‘collaborators’. As soon as the trial outcomes are available, all participating patients will receive a letter with a summary of the outcomes.

## Discussion

The left side of the liver is a common site for and is more seriously affected by hepatolithiasis. Hepatectomy has been the most effective treatment for removing IHD stones and resolving bile duct strictures, thereby decreasing the risk of recurrence [3, 22]. Compared with open hepatectomy, laparoscopic hepatectomy enables more accurate visualization and vascular anatomy. However, only a few retrospective studies comparing OLH and LLH without the ERAS programme have summarized the complications and long-term follow-up dates, such as residual stones and risk of recurrence, which are the main concerns of surgeons [11, 12].

The present study is the first RCT to compare the benefits of the laparoscopic approach to left-sided hepatolithiasis in Chinese patients undergoing hepatectomy with the ERAS programme. It is well recognized that a well-conducted double-blind RCT provides the highest level of evidence to prove the possible benefits of laparoscopic liver resection. However, performing an RCT during surgery is not without difficulties, and alternative trial designs may be necessary [25, 28]. First, the intervention needs to be tested in a standardized environment, and the properties of the intervention should remain unchanged during the trial period. The surgeons should have advanced laparoscopic experience and open liver resection experience. Both the LLS and the ERAS programme-enhanced recovery protocol provide the standardization needed. Second, the intervention should be double-blinded. Although double blinding in a surgical trial is difficult, using a fixed abdominal dressing for 3 days after surgery is feasible and should prevent both ward caregivers and patients from knowing the type of intervention. The trial with randomization of patients and surgeons with treatment equipoise and a prospective registry to cover both surgeons who believe that, based on their laparoscopic experience, randomization is not ethically justified and patients with a strong treatment preference will provide external validity. This trial design, which capitalizes on rather than ignores the differences between patients, will provide more robust outcome data and should lead to continuous performance monitoring after the trial.

Worldwide, the median LOS at a hospital for open and laparoscopic resections varies from 4 to 8 days [6, 9, 12]. The reasons for the delay of discharge and the discharge location are often absent, and to date, a clear definition of recovery has been used in only a few publications. Departing from the standpoint that an RCT should be conducted, a primary question concerns which sample size should be used. In our opinion, a reduction of approximately 1 day in the time to recovery or LOS at a hospital after laparoscopic resection would be a disappointingly low gain. To prove such a reduction, 190 patients are needed (*α* = 0.05 and power of 90%), making the trial unlikely to be reasonably moderate, and it is to be expected that patient accrual will be accomplished within 3–4 years.

This study has several limitations that should be acknowledged. It will be conducted at a single institution. Due to the small sample size, the findings from this trial will not allow for established clinical application but rather will serve to inform the need for larger multicentre RCTs on LLH for OLH.

### Trial status

Recruitment of participants commenced on 10 August 2019 and will be completed in December 2024.

### Supplementary Information

**Additional file 1.** Spirit checklist for *Trials*.

## Data Availability

Individual participant data that underlie the results reported in this article will be published after deidentification. Documents that will be shared further are as follows: study protocol, statistical analysis plan, analytic code, and aggregated individual study data. Routine/administrative data from health insurances will not be made available. Access to data will be provided for anyone legitimately interested in it. Analytic code and aggregated individual study data will be made available on an online repository immediately after publication (or within the peer review process). Participants give informed consent to publish their data after deidentification (except for the routine/administrative data from the health insurances).

## Abbreviations

OLH: Open left-sided hepatectomy
LLH: Laparoscopic left-sided hepatectomy
ASA: American Society of Anaesthesiologists
CRFs: Clinical Report Forms
ISGLS: International Study Group of Liver Surgery
